# A Cross-Sectional Analysis of Blood Spot per- and Polyfluoroalkyl Substances (PFAS) from Adolescents in Chitwan Valley, Nepal

**DOI:** 10.3390/epidemiologia7010005

**Published:** 2026-01-04

**Authors:** Lauren Marie Ward, Shristi Bhandari, Hafsa Aleem, Jaclyn M. Goodrich, Rajendra Prasad Parajuli

**Affiliations:** 1 Department of Environmental Health Sciences, University of Michigan School of Public Health, Ann Arbor, MI 48109, USA; lmward@umich.edu (L.M.W.); hafsaale@umich.edu (H.A.); 2 Central Department of Zoology, Tribhuvan University, Kathmandu 44618, Nepal; shristibhandari23@gmail.com; 3 Herbert Wertheim School of Public Health and Human Longevity Science, University of California, San Diego, La Jolla, CA 92093, USA

**Keywords:** PFAS, per- and polyfluoroalkyl substances, biological monitoring, adolescent health, Nepal

## Abstract

Background/Objectives: Per- and polyfluoroalkyl substances (PFAS) are globally widespread contaminants linked to adverse health outcomes, including immune dysregulation. We aimed to characterize PFAS exposure among adolescents in Nepal. We conducted a cross-sectional study in Chitwan District, Nepal, during September–October 2023, enrolling 73 adolescents from the Chitwan Birth Cohort. Methods: Dried blood spots from 48 participants were analyzed for 45 PFAS by liquid chromatography–tandem mass spectrometry. Sociodemographic and contextual behavioral covariates information (e.g., water source and local fish consumption) were collected via questionnaire. We used linear regression to analyze the association between contextual behavioral covariates and PFAS concentrations. Results: PFOS was detected in 46% of samples, followed by PFNA (25%) and PFOA (12.5%); other PFAS were rarely detected. Participants who consumed locally caught fish more than once per month had significantly higher PFOS levels (β = 0.35, *p* = 0.006). Conclusions: Frequent fish intake was the only factor significantly associated with PFAS levels, suggesting a dietary exposure pathway. This study provides the first documentation of PFAS exposure among Nepalese adolescents, revealing low-level exposures. Findings underscore the need for ongoing surveillance of environmental contaminants in vulnerable populations.

## 1. Introduction

Per- and polyfluoroalkyl substances (PFAS) are a group of synthetic compounds with oil- and water-resistant properties that are used in a variety of consumer products and industrial applications [1]. Children may be exposed to PFAS through their interactions with their environment, food and water consumption, or prenatally [2]. PFAS exposure has been associated with many adverse health impacts, including increased risk for several cancers, reproductive consequences, poor developmental and birth outcomes, and obesity [3]. Additionally, exposure to elevated levels of PFAS in childhood has been associated with reduced immune response to vaccinations [4], impaired antibody production [5], increased risk of infectious disease, and immunosuppression [2]. PFAS exposure in childhood is associated with poor growth and behavioral outcomes [6] and alterations in vaccine-induced immunity and allergic hypersensitivity [4,7].

PFAS have been identified on every continent [8] and are a globally recognized public health issue, as indicated by the 2015 signing of the multinational Madrid Statement calling for “limiting the production and use of PFASs and in developing safer non-fluorinated alternatives” and the inclusion of multiple types of PFAS as persistent organic pollutants (POPs) under the Stockholm Convention [9]. PFAS contamination of drinking water has been documented across the Asian–Pacific area and is well-documented in waterways of India and China and on the Tibetan Plateau [10,11]. The extent of PFAS contamination tends to be greater in industrialized, urban areas than in rural or remote regions of the same country [10,12,13]. While PFAS concentrations, in general, are often reported in the literature to be lower in the environments of low- and middle-income countries [14,15,16], environmental regulation and testing can be severely limited by cost barriers and capacity, leaving gaps in surveillance and research. PFAS concentrations in the environment are predicted to continue to increase with continued urbanization and development [17], posing an increasing risk to human health in urban centers of low- and middle-income countries (LMICs).

Though PFAS are distributed globally, the current literature on PFAS in Nepal is limited. Studies in 2019 and 2021 documented the presence of PFAS in runoff from Mount Everest [18] and in soil, water, and dust in urban and rural regions of the country in a 2019 Center for Public Health and Environmental Development (CEPHED) country-wide report [19]. A 2014 study quantified sixteen PFAS in soils of the Koshi River in southeastern Nepal. This area is rural, hilly, and has little industrialization. Ten of the sixteen PFAS were detected in surface soil samples. Due to the lack of industry in the area, they hypothesized that the PFAS deposits in this region originated from the use and disposal of PFAS-containing consumer products and long-range atmospheric transport [20]. This study aims to assess exposure of Nepali adolescents to PFAS. At the time of writing, this study represents the first documentation of PFAS exposure in Nepali residents.

## 2. Materials and Methods

### 2.1. Study Population and Data Collection

**Figure A1:**
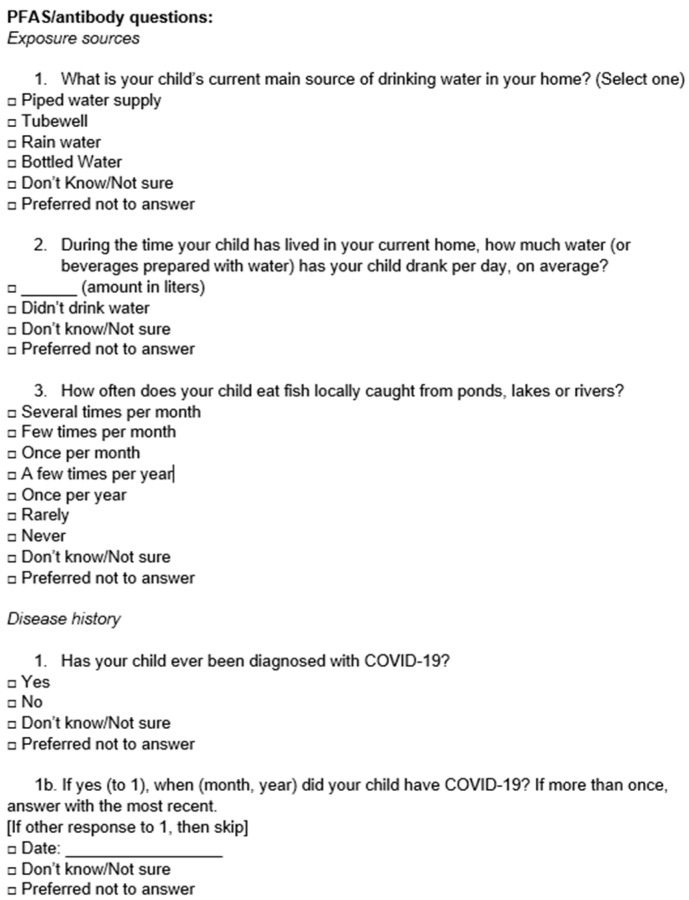
Survey instrument for exposure assessment during home visits.

The original 2008 cohort was recruited using a purposive sampling strategy, with eligible pregnant women enrolled on a first-come, first-served basis at Bharatpur General Hospital until 100 participants provided informed consent, as described in prior publications [21]. Although this analysis is cross-sectional, the participants were originally enrolled as part of the longitudinal Chitwan Birth Cohort established in 2008, allowing retrospective assessment of environmental exposures during adolescence. All 73 participants still living in Chitwan District were contacted via phone call, and home visits were arranged by the study team between September and October 2023. During the home visit, a survey on drinking water source and consumption was orally administered to the participant or a parent in Nepali, and responses were recorded, in English, by the study team. Survey questions were developed based on questions in the “PFAS Exposure Assessment Question Bank: Child” section of the 2018 *Per- and Polyfluoroalkyl Substances Exposure Assessment Technical Tools* publication from the United States Centers for Disease Control and Prevention (CDC) Agency for Toxic Substances and Disease Registry [22]. An English translation of the survey is included in Appendix A (Figure A1). Although the source materials were derived from the CDC’s Child Question Bank, the questionnaire was adapted to be age-appropriate for adolescent participants (~15 years old) and revised linguistically to match local context. The survey was administered directly to participants or, when needed, with parental input. Parents or guardians of all participants provided written informed consent. Study procedures were approved by the Nepal Health Research Council Ethical Review Board (Protocol No. 198/2022P. 12 August 2022) and the University of Michigan Health Sciences and Behavioral Sciences Institutional Review Board (IRB-HSBS) (Study ID: HUM00231173. 3 September 2023). All participants were aged approximately 15 years at the time of survey (born September–October 2008) and were thus classified as adolescents for the purpose of this study.

### 2.2. Dried Blood Spot (DBS) Sample Collection

Capillary blood was collected via finger stick. The participant was asked to apply hand sanitizer, and then the fingertip to be punctured was cleaned with an alcohol prep pad. The fingertip of the index finger of the dominant hand was lanced by a member of the study team with medical laboratory or community health worker training using a BD Microtainer Contact-Activated Lancet (Blue). One blood droplet was applied to each of three out of four circles on a Whatman 903 Protein Saver card. The protein saver cards were dried on a flat surface until the conclusion of the home visit and were then placed into individual plastic bags with a desiccant packet for transportation. At the conclusion of each day’s home visits, cards were placed onto a clean, flat surface to dry for 24 h. The protein saver cards were then sealed into individual plastic bags with a packet of desiccant. Samples were transported from Chitwan District to the University of Michigan (Ann Arbor, MI, USA) in two batches at room temperature. Upon arrival at the university, the bags containing the cards were stored at 4° Celsius (within eighteen days of sample collection) until further processing.

### 2.3. PFAS Testing

**Figure 1:**
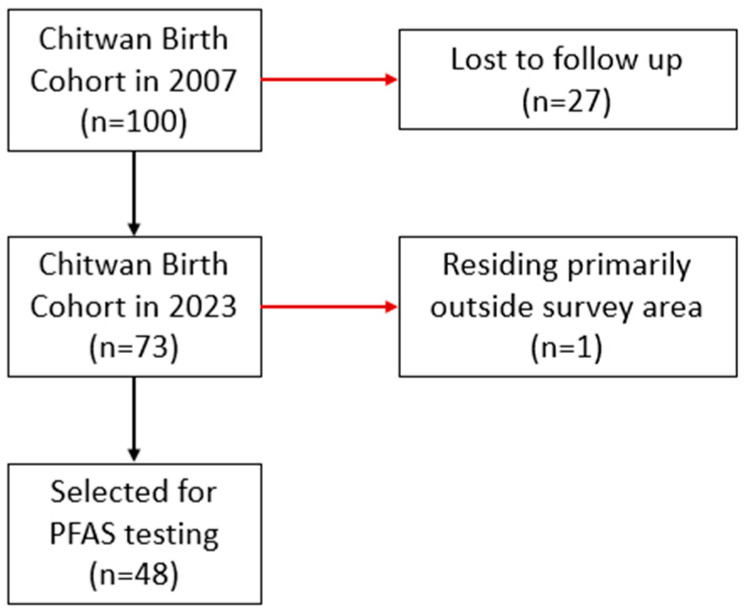
Flow chart of the sample selection process.

A subset of 48 dried blood spot samples was selected for PFAS analysis Eurofins Environmental Testing (Sacramento, CA, USA). These samples were randomly selected from those participants who had a blood sample and complete demographic data. The selection process is detailed in Figure 1. One whole spot per participant was excised from the Whatman 903 Protein Saver card. The spot was quartered and transferred to a 1.5 mL tube. Samples were then shipped on ice to the Eurofins laboratory.

Samples were analyzed for an expanded list of 45 PFAS analytes by Eurofins Environmental Testing (Sacramento, CA, USA) via liquid chromatography–tandem mass spectrometry and online solid-phase extraction using an Exion LC system (SCIEX, Toronto, ON, Canada) with three pumps and an SCIEX 7500 mass spectrometer (SCIEX, Toronto, ON, Canada). This method was established previously by Eurofins laboratories as described in Carignan et al. [23,24,25]. DBS were analyzed using by modifying the protocol for volumetric microsamplers for blood spots, assuming that each blood spot contained 75 µL of whole blood. The 75 µL may be an overestimate; however, it was selected based on previous data that found that DBS often contain a volume greater than a single drop of blood [26,27,28], in combination with manufacturer specifications that each circle on the card can hold 75–80 µL of blood when saturated. Quality control procedures included method blanks, laboratory control samples, and a known standard from (National Institute of Science and Technology (NIST) SRM 1957 Organic Contaminants in Non-Fortified Human Serum). The limit of detection (LOD) for perfluorohexanesulfonic acid (PFHxS), perfluorooctanesulfonic acid (PFOS), perfluorononanoic acid (PFNA), perfluorodecanoic acid (PFDA), and perfluoroundecanoic acid (PFUnDA) was 0.04 ng/mL. The LOD for PFOA was 0.12 ng/mL. The other PFAS analytes that were not detected in this cohort had LODs ranging from 0.04 to 0.18 ng/mL. Due to the low concentrations of PFAS detected, we report the whole blood concentration here. In general, serum PFAS levels are estimated to be twofold higher than whole blood [29]. Values below the LOD were replaced with LOD/√2 prior to analysis, in line with a commonly used imputation technique in environmental epidemiology to reduce bias in left-skewed data distributions.

### 2.4. Statistical Analysis

All statistical analyses were performed in the R Project for Statistical Computing, version 4.3.1 or greater. Descriptive statistics were calculated for covariates including demographic variables and PFAS concentrations in whole blood. A linear model was created to examine the relationship between frequency of local fish consumption and PFOS levels. PFOS was natural-log-transformed to achieve a normal distribution. We evaluated the relationship between frequency of local fish consumption and natural log-transformed whole-blood PFOS concentrations using linear regression models, implemented via the glm() function in R. Group differences in mean PFOS concentrations between consumption categories were assessed using independent-samples *t*-tests.

## 3. Results

The study sample included 73 adolescents in the Chitwan Birth Cohort who participated in home visits between September and October 2023. A subset of 48 participants’ DBS samples was selected for PFAS testing. Table 1 outlines key descriptive characteristics of the population.

All participants in the study were born between September and October 2008 [21]. The mean cohort annual household income of 648,278 Nepali rupees (NPR) was slightly lower than the mean annual income of 718,117 NPR per household in Bagmati Province in the 2022/2023 Nepal Living Standard Survey IV. The distribution of primary drinking water sources in the cohort was similar to the distribution reported in the Nepal Living Standard Survey IV [30]. In the Chitwan Birth Cohort, the largest portion of participants (58%) reported obtaining drinking water from a piped water supply, followed by 37% of participants who reported obtaining drinking water from a tubewell. In Nepal Living Standard Survey IV data, Bagmati Province residents reported mostly obtaining drinking water from a piped source (66%). Significantly more households in the Chitwan Birth Cohort reported obtaining drinking water via tubewell (3%) than Bagmati Province residents, though the proportion of the cohort using a tubewell as a primary drinking source is similar to Nepal’s national proportions. Most participants (64%) reported infrequent consumption of locally caught fish.

PFAS concentrations are reported in Table 2. PFOS was detected most frequently in 46% of participants, while PFNA was detected in 25% of participants, and PFOA was detected in 12.5%. PFOA and PFDA had the highest mean concentrations, though these results should be interpreted with caution due to the use of an estimated volume per sample. Out of 45 PFAS analytes listed in Appendix B (Table A1), only six were detected in at least one sample.

**Figure 2:**
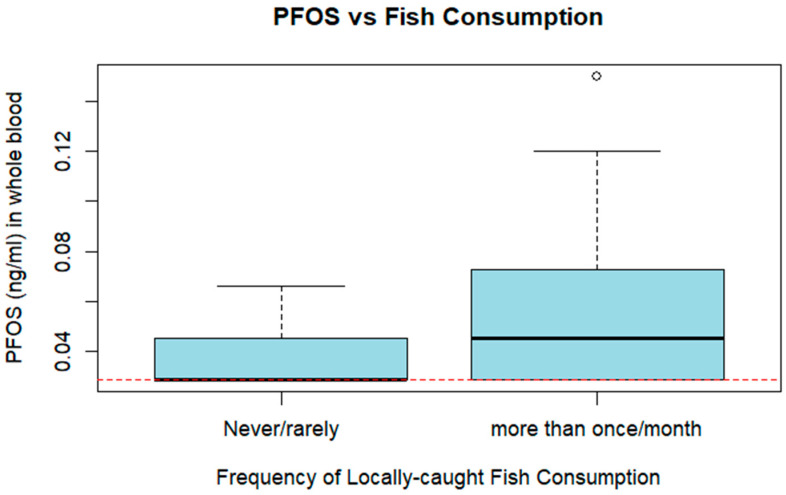
Boxplot of PFOS concentration vs. frequency of fish consumption (*n* = 22). Dotted red line indicates values below the LOD, set at LOD/√2). Mean is indicated by the unbroken black line within the box. Outliers are represented by an unfilled circle.

The mean PFOS concentration in whole blood among those participants reporting regular local fish consumption (more than once a month/≥12 times annually)—0.058 ng/mL (standard deviation: 0.037)—differed significantly from the mean PFOS concentration of those participants reporting consuming local fish “never or rarely” (fewer than 12 times annually)—0.036 ng/mL (standard deviation: 0.016) (*p* = 0.03) via *t*-test (Figure 2). A statistically significant positive association was observed between consuming locally caught fish more than once per month and PFOS, with a beta-coefficient e of 0.35 (standard error: 0.12) (*p* = 0.006).

## 4. Discussion

In this cross-sectional study of adolescents in the Chitwan Birth Cohort, we assessed biomarkers of PFAS exposure. Among 45 PFAS analytes tested for, we identified 6 PFAS present in at least one or more members of a 48-person subset of the cohort. These PFAS were PFHxS, linear PFOS, linear PFOA, PFNA, PFDA, and PFUnDA. PFOS was the most commonly detected analyte, present in 46% of samples. PFNA and PFOA were detected in 25% and 13% of participants, respectively. PFHxS was detected in two participants, and PFDA and PFUnDA were each detected in one participant. The maximum concentration of any PFAS detected was PFOS at 0.15 ng/mL. Though we expect low levels of PFAS exposure in rural areas of Nepal, the whole blood PFAS concentrations reported in this analysis are likely an underestimate of the true concentrations due to the use of dried blood spots and a conservative estimate of blood volume. Previous biomonitoring work has demonstrated that serum PFAS is approximately twofold higher than levels in whole blood [29], corresponding to a maximum PFOS serum level measurement around 0.3 ng/mL in the present study—significantly lower than levels detected by previous studies in North America (28.6 ng/mL) and other countries in Asia (8.5–9.0 ng/mL) [31]. The detected concentrations fell into the lowest tier of PFAS exposure according to a previous study which compiled 2325 publications on human PFAS exposure and found that, globally, PFOS, PFHxS, and PFOA exposure levels were highest. The researchers also noted that exposure to these PFAS had decreased over the past twenty years in some regions, but exposure to other PFAS did not follow a similar trend in other studied areas, such as East Asia. Specifically, serum PFOA demonstrated a slight increasing trend in China and Japan over the past three decades, and PFNA also displayed an increasing trend [31]. This global data, paired with the pilot detection results of this study, highlights the importance of continued monitoring for PFAS which includes varied geographic locations and regions with different degrees of urbanization in order to fully understand the scope of worldwide PFAS exposure.

Consumption of locally caught fish once or more per month was significantly associated with higher PFOS concentrations in whole blood. PFOS is the most commonly detected PFAS in freshwater fish in the United States. While the present study did not directly analyze fish samples for PFAS, the correlation between fish consumption and human PFOS levels, paired with the known bioaccumulation of PFOS in fish and previous detection of PFAS in Nepali waterways, provides some inferential evidence for an exposure pathway via water, which should be more fully explored in further research in this geographic region. The results suggested that the geometric mean PFOS level was 42% higher in participants that consumed local fish. Additionally, this result should be interpreted with caution due to the small sample size and low levels of PFOS detection overall (detected in 46% of participants). A study of freshwater fish analyzed from 2013 to 2015 as part of the United States Environmental Protection Agency’s biomonitoring program suggested that even infrequent consumption of freshwater fish could contribute to an increase in serum PFAS levels [32]. PFOS and PFHxS levels in fish caught in Swiss lakes have been reported to exceed EU limits for safe consumption [33]. Contamination of seafood with PFAS has been recorded in various South Asian countries including Bangladesh, India, and Sri Lanka [34]. Regular fish consumption was uncommon in the Chitwan Birth Cohort, with 33% of participants reporting a meal containing locally caught fish once or more per month. This is reflective of national trends in Nepal; however, we did not survey the participants on imported fish consumption and are likely underestimating total fish consumption. As a landlocked country, fish consumption is lower than in neighboring countries, though annual consumption of fish increased threefold from 1.1 kg 1997 to 3.1 kg in 2017 per capita [35]. In 2012, Nepal’s National Nutrition Program recommended that each person consume 30 g of fish per day to meet nutritional goals [36], though this goal would likely require significant scaling-up of domestic fish production [37]. A 2023 risk assessment of seafood sold in U.S. grocery stores detected PFAS in 74% of samples, with PFHxS detected most commonly. The highest level of PFAS was detected in fish imported to the U.S. from Estonia, while the highest median PFOA levels were detected in clams imported from China [38], highlighting challenges of monitoring both domestically and internationally produced fish to ensure safe consumption. Future investment in the domestic Nepali aquaculture industry presents a unique opportunity to concurrently develop a robust pollutant monitoring program.

The Center for Public Health and Urban Development reported in their 2019 Nepal PFAS Country Situation Report that PFAS had been detected in water, soil, and house dust through several studies in Nepal, including in samples from a rural, agricultural area. Currently, PFAS are unregulated in the country [19]. However, Nepal has signaled plans to prioritize the reduction of environmental contaminants by signing onto the Stockholm Convention on Persistent Organic Pollutants in 2006 and submitting an updated National Implementation Plan that includes PFOS and PFOA in 2017 [19,39]. Regulating PFAS would also contribute to Nepal’s progress toward several United Nations Sustainable Development Goals (SDGs) [40] concerning human health, environmental health, and water pollution.

This study was not without limitations. Most notably, the small sample size reduced statistical power. Since our true blood volume on each spot was not known, we deliberately used a conservative estimate for the volume of blood in each dried blood spot to prevent overestimating PFAS concentrations in this population. Environmental factors during sample collection and transport also introduced potential challenges and contaminants; however, prior research has demonstrated the reliability of dried blood spot analysis under field conditions [23,24,25,41]. Additionally, the dietary survey inquired only about locally caught fish, excluding imported fish, and captured only the frequency—but not the quantity—of fish consumption. The PFAS analyses were conducted on a subset of participants because of financial constraints; however, the subsample was randomly selected to minimize selection bias. Finally, the use of LOD/√2 substitution may introduce some distortion in regression estimates when a large proportion of values fall below the LOD. Despite these challenges, this study successfully employed validated biospecimen collection techniques to document PFAS biomarker profiles among adolescents in Nepal’s Chitwan District. To our knowledge, this represents the first investigation of PFAS burden in human populations within Nepal.

## 5. Conclusions

This pilot study assessed PFAS biomarker concentrations in adolescents from the Chitwan Birth Cohort in Nepal using dried blood spots. Among 45 analytes, PFOS was the most frequently detected, followed by PFNA and PFOA. We observed a significant association between regular consumption of locally caught fish and elevated PFOS concentrations. These findings highlight the presence of PFAS exposure in a rural Nepali population and emphasize the need for future surveillance and regulatory efforts in low- and middle-income countries.

## Figures and Tables

**Table 1:** Demographic characteristics of the Chitwan Birth Cohort.

Variable	Full Cohort (*n* = 73) Mean (SD)	Subset (*n* = 48) Mean (SD)
Age (years)	15.00 (0.03)	15.01 (0.03)
Annual family income (NPR)	648,278 (542,865.10)	670,213 (540,645.70)
Annual family income (USD)	4862 (4071.89)	5027 (4054.84)
Water consumption per day (L)	1.28 (0.79)	1.39 (0.89)
Sex: Male	33 (45.21%)	21 (43.75%)
Sex: Female	40 (54.79%)	27 (56.25%)
Primary drinking water: Piped supply	42 (57.53%)	29 (61.70%)
Primary drinking water: Tubewell	27 (36.99%)	17 (36.17%)
Primary drinking water: Rainwater	0 (0.00%)	0 (0.00%)
Primary drinking water: Bottled water	1 (1.37%)	1 (2.13%)
Fish consumption: Often (>1 time/month)	25 (34.25%)	16 (33.33%)
Fish consumption: Occasionally (≤1 time/month)	15 (20.55%)	10 (20.83%)
Fish consumption: Rarely or never	32 (43.84%)	22 (45.83%)

**Table 2:** Concentrations of detected PFAS.

PFAS	Full Name	Number Above LOD	Percent Above LOD	Mean (SD)	Minimum (ng/mL)	Maximum (ng/mL)
PFHxS	Perfluorohexanesulfonic acid	2	4.17	†	<LOD	0.058
PFOS	L-Perfluorooctanesulfonic acid	22	45.83	0.062 (0.03)	<LOD	0.15
PFOA	L-Perfluorooctanoic acid	6	12.5	†	<LOD	0.34
PFNA	Perfluorononanoic acid	12	25	0.054 (0.01)	<LOD	0.094
PFDA	Perfluorodecanoic acid	1	2.08	†	<LOD	0.1
PFUnDA	Perfluoroundecanoic acid	1	2.08	†	<LOD	0.079

† Means not calculated when % above LOD < 25%. Reported concentrations represent ng/mL in whole blood.

## Data Availability

The raw data supporting the conclusions of this article will be made available by the authors upon reasonable request.

## Abbreviations

The following abbreviations are used in this manuscript:

ATSDR	Agency for Toxic Substances and Disease Registry (USA)
CEPHED	Center for Public Health and Urban Development (Nepal)
EPA	Environmental Protection Agency (USA)
FAO	Food and Agriculture Organization of the United Nations
LMIC	Low- and middle-income countries
LOD	Limit of detection
NIEHS	National Institute of Environmental Health Sciences (USA)
NPR	Nepali rupees
NTP	National Toxicology Program (USA)
PFAS	Per- and polyfluoroalkyl substances
PFDA	Perfluorodecanoic acid
PFHxS	Perfluorohexanesulfonic acid
PFNA	Perfluorononanoic acid
PFOA	L- Perfluorooctanoic acid
PFOS	L-Perfluorooctanesulfonic acid
PFUnDA	Perfluoroundecanoic acid
POPs	Persistent organic pollutants
SDGs	Sustainable Development Goals
USD	United States dollars

## Appendix B

**Table A1:** All PFAS measured and percent of samples above the limit of detection.

PFAS	Full Name	% Above LOD	LOD (ng/mL)
PFHxA	Perfluorohexanoic acid	0	0.08
PFHpA	Perfluoroheptanoic acid	0	0.04
Linear PFOA	L-Perfluorooctanoic acid	12.5	0.12
Branched PFOA	Br-Perfluorooctanoic acid	0	0.12
	Total PFOA	12.5	0.12
PFNA	Perfluorononanoic acid	25	0.04
PFDA	Perfluorodecanoic acid	2.08	0.04
PFUnDA	Perfluoroundecanoic acid	2.08	0.04
PFDoA	Perfluorododecanoic acid	0	0.04
PFTrDA	Perfluorotridecanoic acid	0	0.04
PFTeDA	Perfluorotetradecanoic acid	0	0.04
PFHxDA	Perfluoro-n-hexadecanoic acid	0	0.04
PFBS	Perfluorobutanesulfonic acid	0	0.18
PFPeS	Perfluoropentanesulfonic acid	0	0.04
PFHxS	Perfluorohexanesulfonic acid	4.17	0.04
PFHpS	Perfluoroheptanesulfonic acid	0	0.08
Linear PFOS	L-Perfluorooctanesulfonic acid	45.83	0.04
Branched PFOS	Br-Perfluorooctanesulfonic acid	0	0.04
	Total PFOS	45.83	0.04
PFNS	Perfluorononanesulfonic acid	0	0.04
PFDS	Perfluorodecanesulfonic acid	0	0.04
PFDoS	Perfluorododecanesulfonic acid	0	0.04
5:3 FTCA	5:3 Fluorotelomer carboxylic acid	0	0.077
7:3 FTCA	7:3 Fluorotelomer carboxylic acid	0	0.04
6:2 FTCA	6:2 Fluorotelomer carboxylic acid	0	0.055
8:2 FTCA	8:2 Fluorotelomer carboxylic acid	0	0.045
PFOSA	Perfluorooctanesulfonamide	0	0.04
NMeFOSAA	N-methylperfluorooctane sulfonamidoacetic acid	0	0.04
NEtFOSAA	N-ethylperfluorooctane sulfonamidoacetic acid	0	0.04
4:2 FTS	4:2 Fluorotelomer sulfonic acid	0	0.04
8:2 FTS	8:2 Fluorotelomer sulfonic acid	0	0.04
10:2 FTS	10:2 Fluorotelomer sulfonic acid	0	0.04
GenX	HFPO-DA	0	0.04
9Cl-PF3ONS	9-chlorohexanedecafluoro-3-oxanone-1-sulfonic acid	0	0.04
11Cl-PF3OUdS	11-chloroeicosafluoro-3-oxaundecane-1-sulfonic acid	0	0.04
ADONA	4,8-Dioxa-3H-perfluorononanoic acid	0	0.04
PFECHS	Perfluoro-4-ethylcyclohexanesulfonate	0	0.04
NFDHA	Nonafluoro-3,6-dioxaheptanoic acid	0	0.04
PFMBA	Perfluoro-4-methoxybutanoic acid	0	0.04
PFEESA	Perfluoro(2-ethoxyethane) sulfonic acid	0	0.04
PFO5DA	Perfluoro-3,5,7,9,11-pentaoxadodecanoic acid	0	0.08
PFPE-1	Perfluoroalkylether	0	0.094
Hydro-PS Acid	Hydrogen-substituted Perfluoroheptane sulfonate	0	0.04
6:2 FTUCA	6:2 fluorotelomer unsaturated carboxylic acid	0	0.054
8:2 FTUCA	8:2 Fluorotelomer unsaturated carboxylic acid	0	0.04
